# Angiocentric lymph proliferative disorder (lymphomatoid granulomatosis) in a person with newly-diagnosed HIV infection: a case report

**DOI:** 10.1186/s12879-018-3128-3

**Published:** 2018-05-08

**Authors:** Cecilia T. Costiniuk, Jason Karamchandani, Ali Bessissow, Jean-Pierre Routy, Jason Szabo, Charles Frenette

**Affiliations:** 1 Division of Infectious Diseases and Chronic Viral Illness Service, McGill University Health Centre, Royal Victoria Hospital, Glen Site, 1001 boulevard Decarie, Montreal, QC H4A 3J1 Canada; 2 Research Institute, McGill University Health Centre, Montreal, QC Canada; 3 Department of Pathology, McGill University, Montreal Neurological Institute, 3801 Rue Université, Montréal, QC H3A 2B4 Canada; 4 Department of Radiology, McGill University Health Centre, Montreal, QC Canada

**Keywords:** Angiocentric lymph proliferative disorder, Lymphomatoid granulomatosis, HIV

## Abstract

**Background:**

Angiocentric lymph proliferative disorder (ALPD) is a granulomatous lymphoproliferative condition associated with various primary and secondary immunodeficiency states. ALPD is so rare that its prevalence has not been established. Typically affecting middle-aged adults, this condition is often found in the context of Epstein Bar Virus infection and consists of angiocentric and angioinvasive pulmonary infiltrates. Herein, we present a biopsy-proven case of a patient manifesting with a viral meningoencephalomyelitis-like picture with brain, spinal cord, renal and splenic lesions. The diagnosis was confirmed to be ALPD in the context of newly diagnosed HIV infection.

**Case presentation:**

A 35 year-old homosexual man presented with a 5-week history of headaches followed by a 3-week history of horizontal diplopia, limb weakness and right 6th cranial nerve palsy. Lumbar puncture revealed a lymphocytic pleocytosis, high protein and low glucose. Magnetic Resonance Imaging showed scattered lesions throughout the brain and spinal cord and Computed Tomography of the abdomen and pelvis revealed hypodensities involving the kidneys and spleen. HIV testing was positive, with a viral load of 11,096 copies/mL and CD4 count of 324 cells/μL. Serum Epstein Bar virus PCR was positive with 12,434 copies/ml. Right frontal brain biopsy revealed gray matter containing angiogentric cerebritis with organizing infarction but Epstein Bar Virus-in situ preparations were negative and no viral inclusions were identified. A diagnosis of ALPD (also known as lymphomatoid granulomatosis**)** was made. The patient was initiated on antiretroviral therapy and treated with intravenous rituximab every 3 weeks for 4 cycles and made progressive improvements. By the time of discharge his strength had improved and he was ambulating again although with a walker. Within 2 months, his HIV viral load was suppressed. Magnetic Resonance Imaging of the brain 6 months later revealed interval improvement. At his most recent follow-up, 34 months later, his neurological symptoms had almost completed resolved.

**Conclusion:**

Albeit rare, ALPD should be considered in the differential diagnosis of central nervous system lesions in persons with HIV once common etiologies have been eliminated. Furthermore, ALPD involving the central nervous system may occur in in the absence of documented EBV infection in the central nervous system.

## Background

Angiocentric lymph proliferative disorder (ALPD), formerly known as lymphomatoid granulomatosis, is a member of a group of angiocentric and angiodestructive lesions [1]. Lesions are typically composed of EBV positive B-cells intertwined with reactive T-cells [1–3]. The lungs are the most common sites of involvement, followed by the brain [3–5]. Prognosis is generally very poor, especially for persons with central nervous system involvement, and carries a high mortality rate [3, 4, 6]. Although the majority of cases have been reported in immunocompetent individuals, rare cases have been described in individuals with HIV infection [1–16]. Herein, we present the case of 35-year old man with ALPD as the initial presentation of HIV infection.

## Case presentation

A 35 year-old man was transferred from a peripheral hospital to the Montreal Neurological Hospital (MNH) in March 2015 for the diagnostic work-up of a viral encephalitis. Past medical history was significant for cocaine abuse and high-risk sexual behavior with male partners. Human immunodeficiency virus (HIV) testing two years earlier was negative. The patient had initially presented to a community hospital with a 5-week history of headaches and 3-week history of horizontal diplopia. Upon this first presentation, a non-contrast cerebral CT scan was unremarkable and he was treated as having migraines. While at home, he developed right upper extremity weakness, bilateral lower extremity weakness in addition to fecal and urinary incontinence. The patient also endorsed a 20-pound weight loss over 5 weeks. Prior to transfer to the MNH, he underwent lumbar puncture (LP) and his cerebrospinal fluid (CSF) had demonstrated a lymphocytic pleocytosis at 280 cells (0–10 cells), increased protein at 1.5 g/L (1.55 g/L) and low glucose of 1.7 mmol/L (2.5–4.4 mmol/L) and negative gram stain. Intravenous (iv) acyclovir was started due to suspicion of a viral encephalitis.

Upon arrival at the MNH, the patient was drowsy and confused. He was afebrile and vital signs were stable. Cranial nerve exam demonstrated a right 6th cranial nerve palsy. He also had right upper extremity weakness (4/5) and bilateral lower extremity weakness (3/5 bilaterally). Deep tendon reflexes (DTR) were present at the upper extremities bilaterally but absent in the bilateral lower extremities with upgoing plantar responses. Anal tone was absent. Repeat LP demonstrated a high opening pressure at 42 cm H_2_0), lymphocytic pleocytosis at 130 cells (0–10 cells), high protein (1.55 g/L) and low glucose (2.7 mmol/L). While awaiting results of diagnostic testing, he was empirically treated for *Listeria monocytogenes*, Cytomegalovirus and Herpes Simplex Virus with ampicillin 2 g iv q 4 h and valganciclovir 250 mg iv q12 hours. Gram stain, Auramine stain and India Ink stain were negative.

**Fig. 1:**
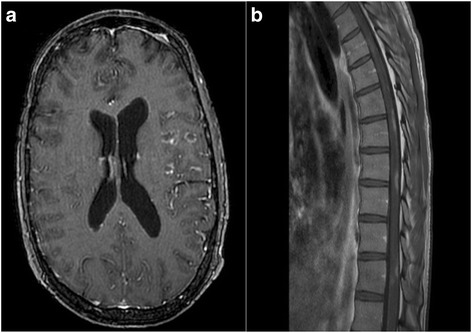
Magnetic Resonance Imaging (MRI) of the brain demonstrated multiple enhancing lesions involving the cerebellum, left midbrain, left pons. A right medullary lesion was also visualized in addition to multiple foci of enhancement involving the cerebral hemispheres (**a**). MRI of the spinal cord showed multiple T2 hyperintensities within the thoracic spine with associated swelling. Faint leptomeningeal enhancement at the anterior cervical cord was also visualized (**b**)

**Fig. 2:**
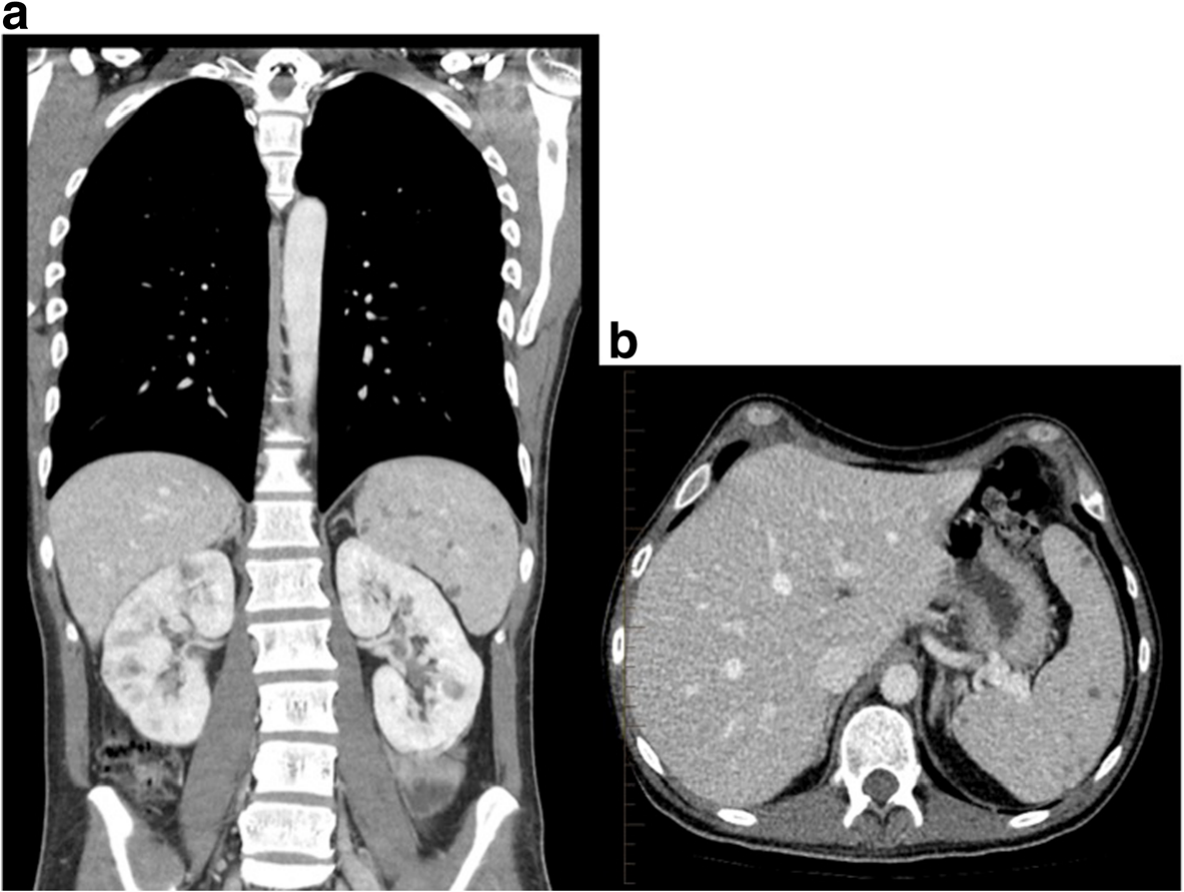
Computed Tomography (CT) of the chest (**a**) and abdomen (**b**) demonstrated left axillary adenopathy, innumerable small hypodensitities in the spleen and multiple lesions in the kidneys

Magnetic Resonance Imaging (MRI) of the brain and whole spine demonstrated multiple enhancing lesions involving the cerebellum, left midbrain, left pons. A right medullary lesion was also visualized in addition to multiple foci of enhancement involving the cerebral hemispheres. There were multiple enhancing lesions in the thoracic spine with associated swelling (Fig. 1). Nerve conduction studies were within normal limits. However, needle studies of the bilateral lower extremities and lumbar paraspinal muscles demonstrated acute denervation suggestive of radicular and or lower motor neuron dysfunction. Computed Tomography (CT) of the chest and abdomen scans revealed left axillary adenopathy, numerable small hypodensitities in the spleen and multiple lesions in the kidneys (Fig. 2). A PET scan was not performed as it would have been unlikely to yield additional insight into the patient’s condition beyond that provided by the MRI.

At admission, peripheral white blood cells count, platelets, haemoglobin, lactate dehydrogenase, liver enzymes and creatinine were normal. C3 and C4 complement were also normal, as was antinuclear antibody screen. Furthermore, tumor markers including alpha fetoprotein, cancer antigen 125 and carcinoembryonic antigen were also normal. Approximately 2–5 days into admission, bacterial CSF and blood cultures were reported as negative. CSF Cryptococcal antigen testing returned negative as did CSF HSV 1 and 2 and serum CMV quantitative PCR. One week into admission, Epstein Bar Virus (EBV) serum viral load came back at 12,434 copies/mL, *Histoplasma capsulatum* serum antigen enzyme immunoassay (EIA) was negative, Lyme disease EIA was negative. JC virus PCR was ordered but due to insufficient quantity of CSF, this test was not performed. Toxoplasmosis immunoglobulin (Ig) G was indeterminate at 2.7 IU/mL and IgM was negative. Serum syphilis EIA was negative. HIV testing then returned positive with a plasma viral load (VL) of 11,096 copies/mL and CD4 count of 324 cells/μL (27%), at which point he was treated with emtricitabine 200 mg, tenofovir 300 mg, darunavir 800 mg and ritonavir 100 mg p.o. daily.

Over the course of the first two weeks, the patient displayed signs of improvement with return of DTR and improvements in strength although he was still incontinent of stool. Furthermore, a trial of spontaneous voiding failed and he required reinsertion of the Foley catheter.

**Fig. 3:**
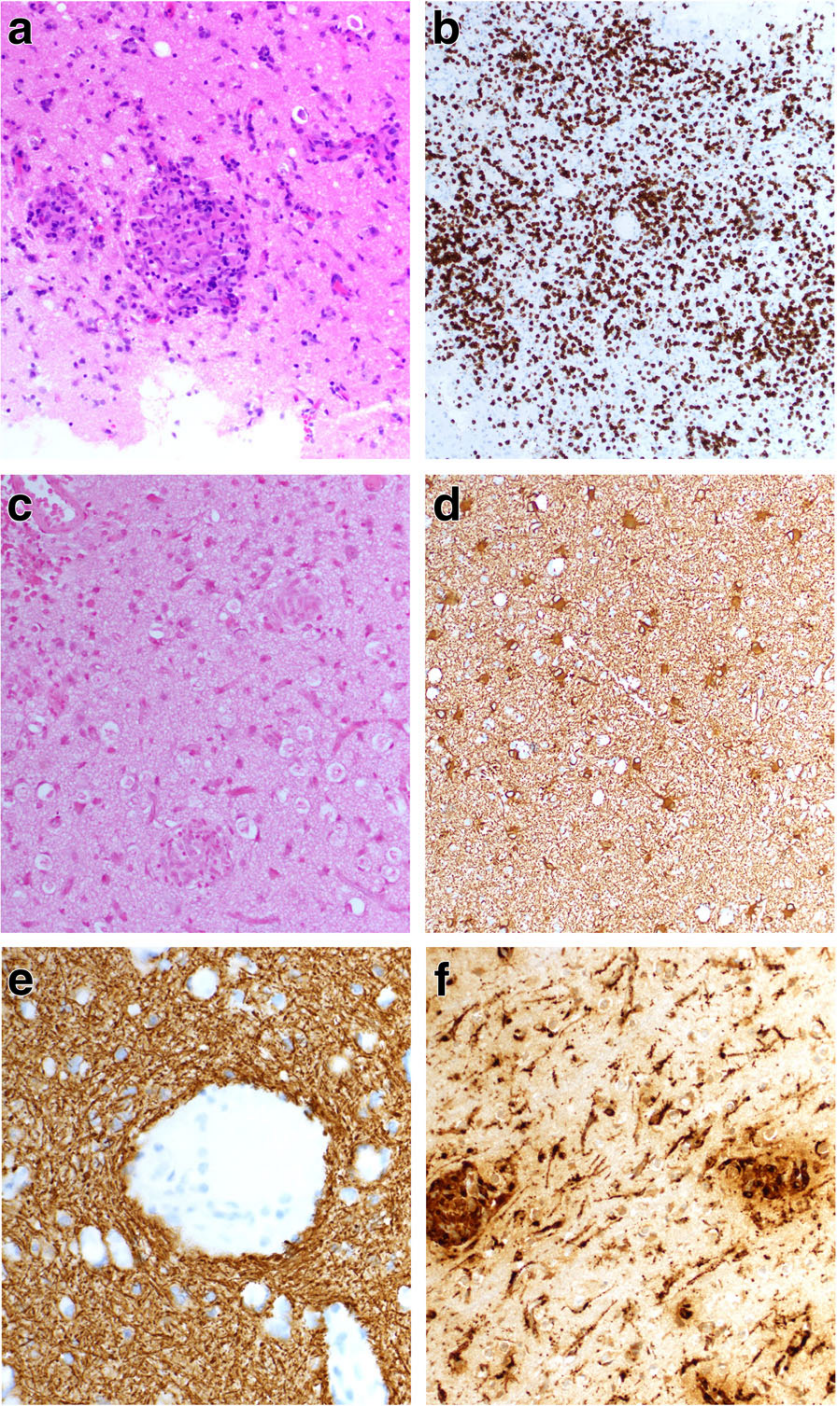
The frontal lobe demonstrated angiocentric granulomas (**a**), CD3 staining demonstrated perivascular and parenchymal T cells (**b**), negative EBV stains (**c**) and reactive astrocytes (**d**). Also shown was a neurofilament with intact axons surrounding granulomas (**e**) and CD68-positive macrophages and microglia (**f**)

During the third week of his admission, the patient underwent brain biopsy of the right frontal lobe. The pathology revealed prominent angiocentric inflammatory patterns, composed of lymphocytes, plasma cells and occasional epithelioid histiocytes. Many of the cortical vessels showed an obliterative endothelialithis. The cortical inflammation overlied and was adjacent to areas of sub-acute organizing infarction. Immunohistochemistry for CD45, CD3 and CD20 confirmed that the majority of the cells were CD3+ and only rare B cells were seen. Most of the lymphocytes were T cells and this finding was suggestive of a reactive process. Immunohistochemistry was performed in both blocks of tissue due to the patchy nature of the pathology. Glial fibrillary acidic protein (GFAP) was lost in areas of infarction. There was concomitant loss of neurofilament, indicating true infarction (as opposed to demyelination). The perivascular lymphoplasmacytic inflammatory infiltrates were composed of primarily CD3 positive T cells with fewer CD20 positive B cells. CD68 showed diffuse microglial activation throughout the cortex and was expressed in the lipidladed macrophages in the regions of infarction. The Ki67 expression was surprisingly low, staining only occasional cells in a perivascular distribution. Stains for both Polyoma virus and toxoplasmosis were negative. There were no viral inclusions and EBV-in situ preparations were also negative. These findings were consistent with grade 1 angiocentric lymph proliferative disorder with a positive EBV PCR in serum (Fig. 3). As the pathology exam yielded the diagnosis, PCR for *Toxoplasmosis gondii* or JC virus DNA were not performed on the brain biopsy. A CT scan was performed following his brain biopsy to ensure no evidence of post-surgical complication. Here it was noted that several of the lesions seen on MRI had partial calcification.

The patient was treated with rituximab 10 mg/kg over 3 weeks for a total of 4 treatments, along with adjunctive steroids. Due to drug interactions, darunavir and ritonavir were replaced by raltegravir 400 mg twice daily. The patient received his first treatment in mid-April 2015 and was discharged 2 weeks later. At the time of discharge, he had a normal mental status. Cranial nerve exam continued to show a right 6th cranial nerve palsy. He also had widespread weakness with upper motor neuron signs, worse in the lower extremities but which had significantly improved. He was also able to ambulate with a walker. Viral load on discharge was 2339 copies/mL and CD4 was 683 cells/μL (CD4 (27%). Fungal cultures of CSF and brain tissue were negative at four weeks and as were Mycobacterial cultures at 12 weeks.

The patient was followed as an outpatient and made progressive improvements. MRI of the head performed in September 2015 showed interval improvement since March 2015 with disappearance of some lesions and reduction of the size of other lesions. No new lesions were identified. At 34 months, his neurological exam and ability to ambulate was normal. However, due to ongoing fatigue he remained off work. His most recent CD4 count in January 2018 was 643 cells/μL (39%) and his HIV viral load has remained undetectable since May 2015.

## Discussion and conclusions

The first sentinel study on ALPD, or lymphomatoid granulomatosis, was published in the early 1970s by Dr. Averill Liebow and described a series of 40 cases of ALPD [5, 17]. At that time, it was known that ALPD was a unique condition which shared many features with Wegener’s granulomatosis and lymphoma [5]. Involving predominantly extra-nodal sites, ALPD is considered to be a lymphoproliferative disorder [18]. Individuals most often affected are those with underlying immunodeficiencies, either hereditary or acquired [18]. Evolution in the techniques used in the field of pathology occurred over the decades. In the mid 1990s, immunohistochemical staining of paraffin-embedded formalized-fixed tissue enabled confirmation of the presence of clonal B cells and reactive T lymphocytes in a majority of the cases previously described [5]. Today, ALPD is considered a large B-cell lymphoma but distinct from Diffuse Large B-cell Lymphoma (DLBL) [4, 8, 19].

In 2010, *Katzenstein* et al. reviewed the cases of ALPD over 4 decades, and examined the 8 largest reported case series ranging in size from 11 to 152 cases [5]. Less than 20 case have been HIV-associated [1–16]. The lungs are the sites most often involved and contain bilateral infiltrates or nodules. Pulmonary nodules may display central necrosis and cavitation [5]. The central nervous system, notably the brain, is the second most commonly involved site although both central and peripheral nervous system involvement may occur. Other commonly involved sites include kidneys, liver and skin. Lymph nodes and spleen are rarely involved [4, 5, 8, 19]. The most common clinical symptoms, experienced by more than half of patients, are cough and fever (> 50%). Rash or nodular skin lesions (40%), malaise and weight loss (35%), neurological symptoms (30%), dyspnea (30%) and chest pain (15%) are also prevalent features [4, 5, 8, 19].

Various radiological features of ALPD may be found. With lung involvement, radiological findings may include pulmonary nodules, cavities, cysts and small pleural effusions [10, 20]. Mediastinal adenopathy is a common finding in over half of patients. However, there are no radiological features of ALPD which are pathognomonic of the condition [10, 20]. With brain involvement, cerebral lesions on CT may consist of multiple, diffusely enhancing lesions in the supratentorial parenchyma [6, 14] which may require intravenous contrast to be visualized [6]. In patients with advanced HIV/Acquired Immune Deficiency Syndrome, Toxoplasmosis and primary CNS lymphoma are the most common causes of ring-enhancing lesions [6]. Moreover, these diseases frequently involve the basal ganglia [6]. Lesions are usually unifocal, situated in the periventricular, temporal, tempoparietal and posterior fossa [6, 21]. These may appear as solid tumor masses or leptomeningeal and parenchymal infiltrates with edema and mass effect [6, 22].

Tissue is required to make a diagnosis of ALPD. Classical histological findings include infiltrates with significant necrosis, usually with a few atypical large B cells in a pleomorphic background of lymphocytes, plasma cells and histiocytes. The infiltrate is characterized pathologically by the accumulation of varying numbers of T cells with variable numbers of atypical clonal EBV-positive B cells which are angiocentric and angioinvasive in a polymorphous inflammatory background [19, 23]. Usually, the large atypical B cells represent a neoplastic component and show evidence of EBV infection [19, 24]. The condition is sub-classified using a grading system based on the number of EBV-positive large B-cell malignant cells. As established by the World Health Organization (WHO) in 2008, three grades of ALPD exist: Grade 1: Large transformed cells are rare (EBV < 5/HPF); Grade 2: Occasional large cells with small clusters (EBV 5–20/HPF); Grade 3: Large aggregates of Reed Sternberg (RS)-like cells (EBV positive cells > 50 HPF). Grading is important to distinguish grade 3 (DLBCL) from grades 1–2 [19], which differ in prognosis. Lower-grade ALPD occasionally undergoes spontaneous remission and is best managed with strategies designed to enhance the host’s underlying immune system. In contrast, high-grade ALPD is best managed by combination chemo-immunotherapy but has inferior outcomes [23].

Our patient had newly diagnosed HIV infection and he had undergone negative HIV testing 2 years prior to his presentation. Due to the rare nature of ALPD and limited number of cases in HIV-infected persons, it is unclear whether symptoms in HIV-infected individuals differ from those without HIV. Similarly, due to the overall small number of cases involving HIV-infected persons, it is unknown whether the incidence or clinical presentation of ALPD has changed since the widespread use of HIV antiretroviral therapy in the mid-1990s [3]. The fact that our patient’s CD4 count was 324 cells/μL suggests that he was not severely immunosuppressed. There is one case report which describes ALPD in the context of HIV immune reconstitution inflammatory syndrome (IRIS), which refers to the paradoxical deterioration in the context of improving CD4 count following antiretroviral treatment initiation [3]. In our patient, HIV CSF viral load was not performed since the association between HIV CSF level and cognitive disturbances are not clearly defined [25].

Interestingly, our patient had a high serum EBV viral load but his brain pathology did not display EBV inclusion. This finding may have occurred since the quantity of EBV in the brain was below the level of detection. EBV RNA detected by in situ hybridization is also helpful in establishing the diagnosis. However, sampling error accounts for some of the EBV-negative cases, especially when small biopsies are taken and where patchy EBV positivity could be missed. Furthermore, it is controversial if truly EBV-negative cases of ALPD exist [23]. Like EBV, it has been suggested that HIV itself may cause cellular transformation [2, 6, 10]. Our patient also had a history of cocaine use, similar to the patient reported by Wyen et al. [3]. Whether these patients’ cocaine use contributed to their to their development of ALPD is unknown.

Brain biopsy may be a necessary tool in the diagnosis of difficult-to-diagnose clinical syndromes. As discussed by *Gilkes* et al.*,* brain biopsies are especially important for disorders requiring disease-specific therapy which may be toxic, in cases of rapid neurological deterioration and for prion disease diagnosis [26]. Brain biopsies are also especially helpful in cases of CNS lesions in immunosuppressed populations, dementias without a diagnosis, angiogram-negative cerebral vasculitis in addition to cryptogeneic neurological diseases [26]. However, there are no clear guidelines to facilitate the selection of the patient, time or location for performing a brain biopsy [26].The optimal treatment strategy for ALPD is unknown, although steroids, cyclophosphamide, interferon alpha-2b and monoclonal antibodies have all been used [5, 10]. The role rituximab played towards the clinical improvement observed in our patient is unclear, since he began to exhibit some neurological improvement even before rituximab was started. It is also possible that our patient’s HIV antiretroviral therapy, which was commenced prior to rituximab, contributed to his recovery. The natural course of ALPD is variable and spontaneous remissions have been reported [3, 4]. However, generally the prognosis is very poor, with mortality rates ranging between 38 and 71% [5]. Therefore, albeit rare, ALPD should be considered in the differential diagnosis of CNS lesions in HIV-infected patients and even in the absence of detectable EBV infection.

## Abbreviations

ALPD: Angiocentric Lymph proliferative disorder
CNS: Central nervous system
CSF: Cerebral spinal fluid
CT: Computed tomography
DLBL: Diffuse Large B-cell Lymphoma
DTR: Deep tendon reflexes
EBV: Epstein Barr virus
EIA: Enzyme immunoassay
HIV: Human immunodeficiency virus
HPF: High power field
IRIS: Immune reconstitution inflammatory syndrome
iv: intravenous
LP: Lumbar puncture
MNH: Montreal Neurological Hospital
MRI: Magnetic resonance imaging
PCR: Polymerase chain reaction
RNA: Ribonucleic acid
RS: Reed Sternberg
WHO: World Health Organization

## References

[1] Gold JE, Ghali V, Gold S, Brown JC, Zalusky R (1990). Angiocentric immunoproliferative lesion/T-cell non-Hodgkin's lymphoma and the acquired immune deficiency syndrome: a case report and review of the literature. Cancer.

[2] Anders KH, Latta H, Chang BS, Tomiyasu U, Quddusi AS, Vinters HV (1989). Lymphomatoid granulomatosis and malignant lymphoma of the central nervous system in the acquired immunodeficiency syndrome. Hum Pathol.

[3] Wyen C, Stenzel W, Hoffmann C, Lehmann C, Deckert M, Fatkenheuer G (2007). Fatal cerebral lymphomatoid granulomatosis in an HIV-1-infected patient. J Inf Secur.

[4] Katzenstein AL, Carrington CB, Liebow AA (1979). Lymphomatoid granulomatosis: a clinicopathologic study of 152 cases. Cancer.

[5] Katzenstein AL, Doxtader E, Narendra S (2010). Lymphomatoid granulomatosis: insights gained over 4 decades. Am J Surg Pathol.

[6] George JC, Caldemeyer KS, Smith RR, Czaja JT (1993). CNS lymphomatoid granulomatosis in AIDS: CT and MR appearances. AJR Am J Roentgenol.

[7] Mittal K, Neri A, Feiner H, Schinella R, Alfonso F (1990). Lymphomatoid granulomatosis in the acquired immunodeficiency syndrome. Evidence of Epstein-Barr virus infection and B-cell clonal selection without myc rearrangement. Cancer.

[8] Colby TV (1989). Central nervous system lymphomatoid granulomatosis in AIDS?. Hum Pathol.

[9] Dominguez-Duran E, Luque-Marquez R, Fontillon-Alberdi M, Abrante-Jimenez A (2011). Laryngeal lymphomatoid granulomatosis in a HIV patient. Enferm Infecc Microbiol Clin.

[10] Gray TC, van Wyk AC, Goussard P, Gie RP (2013). Lymphomatoid granulomatosis: a rare cause of cavitatory lung disease in an HIV positive child. Pediatr Pulmonol.

[11] Haque AK, Myers JL, Hudnall SD, Gelman BB, Lloyd RV, Payne D, Borucki M (1998). Pulmonary lymphomatoid granulomatosis in acquired immunodeficiency syndrome: lesions with Epstein-Barr virus infection. Mod Pathol.

[12] Ioachim HL (1989). Lymphomatoid granulomatosis versus lymphoma of the brain and central nervous system in the acquired immunodeficiency syndrome. Hum Pathol.

[13] Issakhanian M, Chang L, Cornford M, Witt M, Speck O, Goldberg M, Ernst T (2001). HIV-2 infection with cerebral toxoplasmosis and lymphomatoid granulomatosis. J Neuroimaging.

[14] Kapila A, Gupta KL, Garcia JH (1988). CT and MR of lymphomatoid granulomatosis of the CNS: report of four cases and review of the literature. AJNR Am J Neuroradiol.

[15] Pastrana Delgado J, Conchillo Armendariz MA, Vega Vazquez F, Beloqui Ruiz O (1998). Pulmonary lymphomatoid granulomatosis associated with AIDS. Report of a case and review of the literature. An Med Interna.

[16] Schalper KA, Valbuena JR (2011). Primary cerebral lymphomatoid granulomatosis in a HIV-positive patient. Case report. Rev Med Chil.

[17] Liebow AA, Carrington CR, Friedman PJ (1972). Lymphomatoid granulomatosis. Hum Pathol.

[18] Matynia AP, Perkins SL, Li D (2018). Lymphomatoid granulomatosis in a 14-year-old boy with trisomy 21 and history of B-lymphoblastic leukemia/lymphoma. Fetal Pediatr Pathol.

[19] Tissues SS, Campo E, Harris NL, World Health Organization (2008). Classification of Tumours of Haematopoietic and lymphoid.

[20] Lee JS, Tuder R, Lynch DA (2000). Lymphomatoid granulomatosis: radiologic features and pathologic correlations. AJR Am J Roentgenol.

[21] Kerslake R, Rowe D, Worthington BS (1991). CT and MR imaging of CNS lymphomatoid granulomatosis. Neuroradiology.

[22] Budka H (1986). Multinucleated giant cells in brain: a hallmark of the acquired immune deficiency syndrome (AIDS). Acta Neuropathol.

[23] Pittaluga SWW, Jaffe ES (2008). Lymphomatoid granulomatosis. WHO classification of tumours of haematopoietic and lymphoid tissues.

[24] Katzenstein AL, Peiper SC (1990). Detection of Epstein-Barr virus genomes in lymphomatoid granulomatosis: analysis of 29 cases by the polymerase chain reaction technique. Mod Pathol.

[25] Nightingale S, Winston A (2017). Measuring and managing cognitive impairment in HIV. AIDS.

[26] Gilkes CE, Love S, Hardie RJ, Edwards RJ, Scolding NJ, Rice CM (2012). Brain biopsy in benign neurological disease. J Neurol.

